# Unexpected Demography in the Recovery of an Endangered Primate Population

**DOI:** 10.1371/journal.pone.0044407

**Published:** 2012-09-17

**Authors:** Karen B. Strier, Anthony R. Ives

**Affiliations:** 1 Department of Anthropology, University of Wisconsin-Madison, Madison, Wisconsin, United States of America; 2 Department of Zoology, University of Wisconsin-Madison, Madison, Wisconsin, United States of America; Université de Sherbrooke, Canada

## Abstract

Assessments of the status of endangered species have focused on population sizes, often without knowledge of demographic and behavioral processes underlying population recovery. We analyzed demographic data from a 28-year study of a critically endangered primate, the northern muriqui, to investigate possible changes in demographic rates as this population recovered from near extirpation. As the population increased from 60 to nearly 300 individuals, its growth rate declined due to increased mortality and male-biased birth sex ratios; the increased mortality was not uniform across ages and sexes, and there has been a recent increase in mortality of prime-aged males. If not for a concurrent increase in fertility rates, the population would have stabilized at 200 individuals instead of continuing to grow. The unexpected increase in fertility rates and in adult male mortality can be attributed to the muriquis’ expansion of their habitat by spending more time on the ground. The demographic consequences of this behavioral shift must be incorporated into management tactics for this population and emphasize the importance of understanding demographic rates in the recovery of endangered species.

## Introduction

With nearly half of the world’s primates now threatened with extinction, the need for accurate assessments of remaining populations is more urgent than ever [1], [2]. Yet despite the importance of demographic data for setting conservation priorities and for developing informed management tactics, analyses of the underlying processes that regulate population growth are rare [3]–[7]. Although small primate populations may grow when they and their habitats are well protected, whether these populations can persist over the long-term is not yet known [8]–[10]. This uncertainty is especially problematic for forest-dwelling primates with minimal or no dispersal opportunities as a result of habitat fragmentation. In such isolated refuges, population growth may create demographic stresses, making it difficult to distinguish healthy, recovering populations from those that are vulnerable or require active management [11], [12].

Here, we investigate the demographics of one population of the critically endangered northern muriqui using data from a 28-year field study initiated in 1983 at the Reserva Particular do Patrimônio Natural Feliciano Miguel Abdala, a 957 ha forest in Minas Gerais, Brazil (19°50’S, 41°50′W). Fieldwork was conducted with permission from the Conselho Nacional de Desenvolvimento Científico e Tecnológico (CNPq) and the Sociedade para a Preservação de Muriqui, with long-term collaboration from S.L. Mendes and in compliance with all Brazilian and U.S. regulations. The initial population size was estimated to be about 60 individuals; by 2010 the population had reached 288, representing nearly one-third of the entire species [13], [14]. Life-history and behavioral information was collected systematically for one of the two original mixed-sex groups in the population, and in 2003 monitoring expanded to all individuals in the population. Biographical and fertility data were compiled based on known and inferred birth and survivorship records [15]; these data form the basis for our analyses of demographic processes (See Materials and Methods). The non-aggressive, egalitarian social relations that distinguish northern muriqui society eliminate the confounding effects of dominance rank on life history parameters found in other primates living in multi-male, multi-female groups [16]. Female muriquis typically disperse from their natal groups at 5–7 years of age, prior to the onset of puberty [17], but because of the isolation of the forest there was no dispersal; thus, all population change could be attributed to births and deaths. Because the area of the Reserve has not changed over the course of the study period, population size and density are considered to be equivalent. It is possible that the resources available to muriquis have changed through time as both forest regeneration and deterioration from edge effects have been ongoing; however, we have no quantitative measures of the historical changes in resources over the duration of our study period.

We first performed detailed analyses of fertility, survival, and birth sex ratio (BSR) to determine how these demographic rates have changed through time as the population has recovered. Temporal changes in vital rates could be caused by changes in the density of the population or by other factors, such as changes in the behaviors of individuals within the population. We model changes in vital rates in terms of time because we did not want to ascribe an a priori explanation for these changes; by focusing on changes in vital rates over time, we do not exclude the possibility of density dependence, but we also do not exclude other factors.

We use the time-varying vital rates to build a time-dependent demographic projection matrix [18]. We use the projection matrix retrospectively to understand the demographic changes underlying increases in the muriqui population from 1983 to present. Our results show that an unexpected increase in fertility was responsible for the continued increase in population size. Furthermore, an unexpected increase in mortality, especially of prime-aged adult males, suggests demographic stresses on the population possibly imposed by the hyper-inflated fertility rate. Finally, we use the demographic projection matrix prospectively to predict future population growth under the assumptions that fertility rates are the same as they were in 1983 or, for comparison, 2010.

## Results

### Demographic Rates

Demographic analyses of fertility rates, survival, and BSR were performed using Generalized Linear Mixed Models (GLMMs) that can incorporate correlated (non-independent) patterns in the data [19]. In particular, the GLMMs allowed us to incorporate year-to-year variation that affects all age classes similarly; in our analyses of fertility and BSR, the GLMMs allowed us to incorporate individual-to-individual variation (repeated measures). Furthermore, the detailed, individual-level data allowed us to estimate changes in vital rates through time for different age classes.

Our data were used, along with data from 6 other species, in comparative analyses that similarly used GLMMs to estimate vital rates [20]. These comparisons necessitated a least-common-denominator model that could be applied to all data sets. Our GLMMs differ from those in Morris and colleagues [20] in that (i) we use finer age classes; (ii) we analyze survivorships for all age classes simultaneously (while still allowing differences in survivorships among age classes); and (iii) we allow vital rates to change through time (which were observed clearly in the data before statistical analyses).

**Figure 1:**
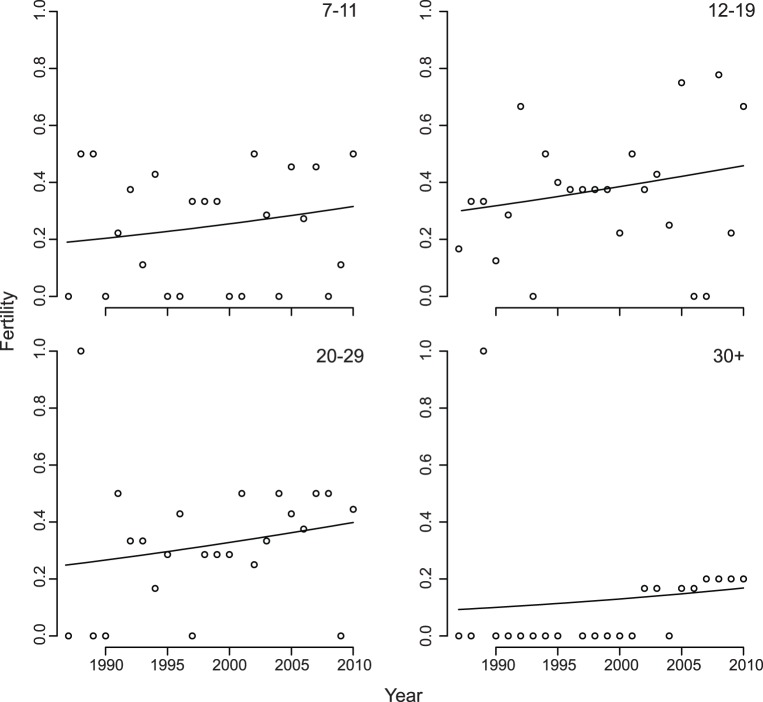
Increase in fertility through time for age groups 7–11, 12–19, 20–29, and 30+ estimated from the Fertility data set of northern muriquis at the Reserva Particular do Patrimônio Natural Feliciano Miguel Abdala, Brazil. Based on 506 observations (mother-years) of 43 mothers from 1987 to 2010.

We estimated time-varying fertility rates for 43 females that were observed continuously for at least a year in the period 1987–2010. As the population size increased by 5-fold from 1983 to 2010, the fertility rate did not decrease as would be expected if there were density-dependent resource limitation. Instead, fertility rates increased through time (*N* = 43 mothers, *P* = 0.049, Table 1, Figure 1). The analyses revealed no statistically significant individual-to-individual variation in fertility rates or year-to-year variation after accounting for the overall increase in fertility rates through time.

**Table 1:** Effect of time on fertility of northern muriquis at the Reserva Particular do Patrimônio Natural Feliciano Miguel Abdala, Brazil, from 1987 to 2010 (equation 1).

Parameter	Value	z-score	P
β_1_	0.030	2.01	0.049
α_7–11_	−1.20		
α_12–19_	−0.59	2.41^1^	0.016^1^
α_20–29_	−0.84	1.37^1^	0.065^1^
α_30+_	−2.02	−2.01^1^	0.045^1^

1 Statistical difference from α_7–11_. *N* = 506 observations (mother-years) from 43 mothers over 24 years. There was no statistically significant variation among females in fertility (σ^2^_m_ = 0, χ^2^_0_+χ^2^_1_ = 0, P>0.5) or among years (σ^2^_y_ = 0.056, χ^2^_0_+χ^2^_1_ = 1.24, P>0.5).

**Figure 2:**
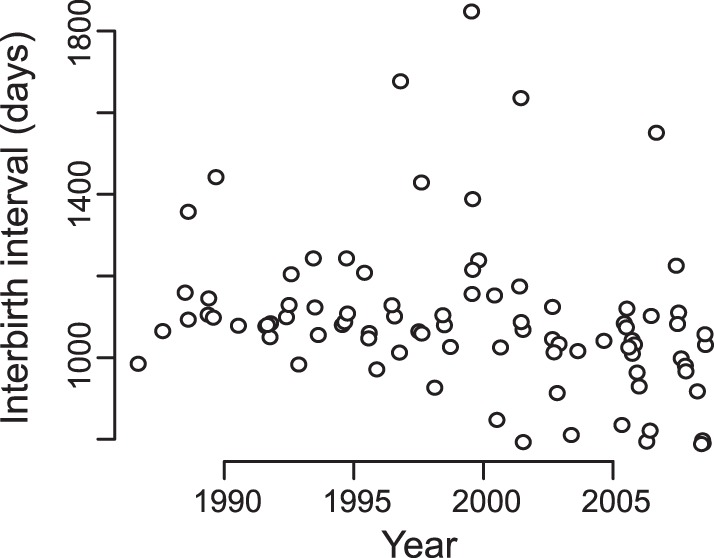
Decrease in interbirth intervals through time estimated from the Fertility data set of northern muriquis at the Reserva Particular do Patrimônio Natural Feliciano Miguel Abdala, Brazil. Based on 88 observations (mother-years) of 28 mothers from 1987 to 2010.

The increase in fertility rates was driven by a reduction in the mean interval between surviving infant births (Figure 2). Including only those interbirth intervals in which the first infant survived for the calendar year of its birth, the interbirth interval dropped from an average of 1201 days to an average of 1015 days from 1987 to 2010 (*N* = 88 births to 28 mothers, *P* = 0.007, Table 2). Interbirth interval did not depend on maternal age, and there was no individual-to-individual variation among females. The effect of the sex of the first offspring was not significant, although it was nearly so (β_2_ = −72.7, P = 0.065, Table 2); on average, the interbirth interval when the first offspring was male was 1044 days, while it was 1137 for females. The significantly shorter parturition-to-conception intervals presumably corresponded to earlier weaning ages. This suggests conditions of improved maternal energetics and nutrition despite the expectation of greater feeding competition for limited food resources as population size increased [21].

**Table 2:** Effect of time on interbirth intervals for northern muriquis at the Reserva Particular do Patrimônio Natural Feliciano Miguel Abdala, Brazil from 1987 to 2010 (equation 2).

With β_2_ removed				With β_2_ included		
Parameter	Value	t-score	P	Value	t-score	P
β_0_	1903			1796		
β_1_ (year of first birth)	−0.0233	2.78	0.0068	−0.019	−2.28	0.025
β_2_ (sex of first infant)				−72.7	−1.88	0.065

*N* = 88 observations from 28 mothers over 24 years when β_2_ is removed; *N* = 87 when β_2_ is included because the sex of one infant was unknown. Mother age (α_age[*i*]_, LLR: χ^2^_2_ = 1.0, P>0.15, with β_2_ removed) and mother-to-mother variation (σ^2^_m_ = 0, χ^2^_0_+χ^2^_1_ = 0, P>0.5, with β_2_ removed) were not statistically significant.

For all interbirth intervals, including those in which the first offspring died within its first calendar year, there were 119 interbirth intervals observed for 30 females; 31 intervals involved the death of the first infant. The interbirth interval was shorter by an average of 243 days if the first offspring died in its first year (t_114_ = −5.59, P<0.0001). This did not affect our estimates of fertility in population projections, however, because these are based on the direct estimates of fertility (Table 1).

A similar analysis of the age of first reproduction (AFR) showed the mean AFR was 8.82 years and ranged from 7.00 to 12.36 years. There was no effect of time, measured as either the mother’s or the first-born offspring’s birthdates, on AFR (P>0.3 and P>0.9, respectively). Three females in the Fertility data set did not disperse from their natal group, and their average AFR was 7.78 years, a year earlier than dispersing females; this difference, however, was not statistically significant (P>0.24).

In contrast to fertility rates, there were significant decreases in survival and in the proportion of female births, both of which will decrease the rate of population growth. We analyzed mortality using records from 463 individuals whose fate was known over the study period (1983–2010). Mortality was statistically modeled (equation 3) as a binomial process in which the probability of dying in a given year *t* was *p_i_* = logit^–1^(*b*_0,age[*i*]_ + *b*_1,age[*i*]_ *t* + *b*_2,age[*i*]_ *t* ^2^+ *y*_year[*i*]_). In the GLMM, the coefficients *b*_0,age[*i*]_, *b*_1,age[*i*]_, and *b*_2,age[*i*]_ are “random effects” [19]. Thus, the term *b*_0,age[*i*]_ can be envisioned as 15 separate coefficients selected from a Gaussian distribution, with each coefficient giving the intercept for one of the 15 age classes. The term *b*_1,age[*i*]_ *t* gives the interaction between age (15 classes) and time (year of study), and the term *b*_1,age[*i*]_ *t* ^2^ gives the interaction between age and time-squared; the latter accounts for possible quadratic (nonlinear) effects of time on mortality. Finally, the random effect *y*_year[*i*]_ accounts for year-to-year variation in mortality affecting all age classes.

**Figure 3:**
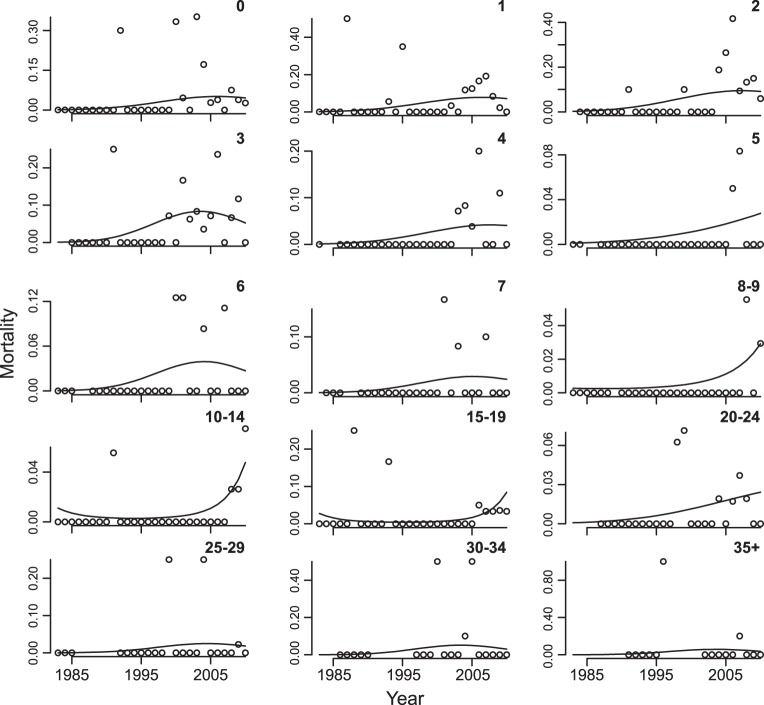
Age-dependent changes in mortality (both sexes) from 1983 to 2010 for northern muriquis at the Reserva Particular do Patrimônio Natural Feliciano Miguel Abdala, Brazil, fitted with **equation 3** (**Table 3**). Each panel gives the mortality for one of the 15 age classes.

**Figure 4:**
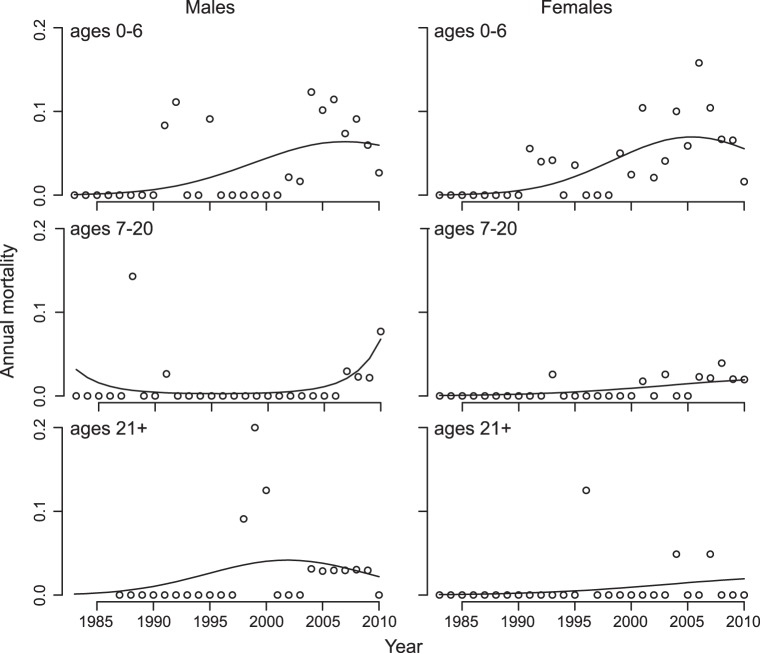
Sex-specific patterns of mortality among age groups for northern muriquis at the Reserva Particular do Patrimônio Natural Feliciano Miguel Abdala, Brazil from 1983–2010. The mortality rate increases through time for all age groups and both sexes, although in a nonlinear fashion. For males, the nonlinear dependence of mortality on time differs significantly among age groups (P = 0.016), with prime-aged males only recently experiencing rapidly increasing mortality; this is not the case for females (P>0.20). For the statistical analyses, age groups were divided more finely than as presented in this figure (Table 3, Figure 3).

There was a statistically significant increase in mortality through time (Table 3). In addition, there was a quadratic effect of time that differed statistically among age classes (Table 3). Inspection of the data reveals that there is an initial rise in mortality for most of the younger (0, 1, 2, 3, 4, 6, 7) and older (25–29, 30–34, 35+) age classes (Figure 3), consistent with resource limitation [4]. Mortality increased but only more recently for the prime-aged adult age classes (8–9, 10–14, 15–19, 20–24, but also the younger age class 5). The pattern of more recent increases in mortality was especially strong in males (Table 3, Figure 4); this difference between sexes suggests that factors other than or in addition to resource limitation were involved in the recent increase in mortality. Finally, there was statistically significant year-to-year variation in mortality that affected all age classes (Table 3).

**Table 3:** Effect of time on age-specific mortality for northern muriquis at the Reserva Particular do Patrimônio Natural Feliciano Miguel Abdala, Brazil from 1983 to 2010 (equation 3).

	Both sexes			Males			Females		
Parameter	Value	Score^1^	P	Value	Score^1^	P	Value	Score^1^	P
β_0_	−4.32			−4.32			−4.44		
β_1_ (linear time trend)	0.137	3.86	0.0001	0.120	2.65	0.008	0.175	2.82	0.005
β_2_ (quadratic time trend)	−0.004	−1.0	0.32	0.001	−0.31	0.75	−0.008	−1.39	0.17
σ^2^_0_	1.11			0.77			1.0		
σ^2^_1_ (variation in *b*_1_ among age classes)	0.004			0.0078			0.0008	0.04	0.42
σ^2^_2_ (variation in *b*_2_ among age classes)	0.00011	8.3	0.006	0.00009	6.4	0.016	0.00004	0.44	0.25
σ^2^_y_ (year-to-year variation)	0.17	4.8	0.015	0.050	0.04	0.8	0.23	2.58	0.06

1 For fixed effects this is the z-score, and for random effects this is the appropriate (χ^2^_0_+χ^2^_1_)/2 or (χ^2^_0_+2χ^2^_1_+χ^2^_2_)/4 score. Statistical tests are only provided for those quantities of interest. For both sexes together, *N* = 686 observations, with 15 age classes and 28 years. For males, *N* = 286 observations, with 12 age classes and 28 years. For females, *N* = 315 observations, with 13 age classes and 28 years. For males and females, age classes were merged so that each age class-year had an observation.

**Figure 5:**
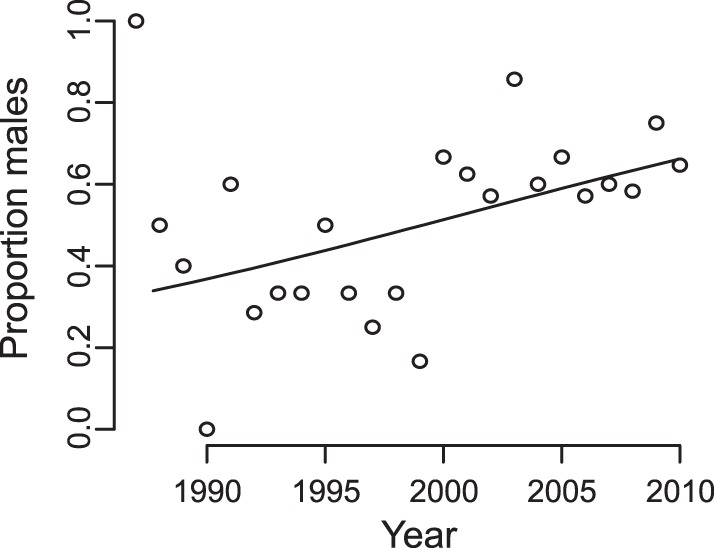
Increase in the BSR through time from the Fertility data set (**equation 4**: z = –2.15, P = 0.032; *N* = 150 observations from 37 mothers over 24 years in 4 age classes).

We investigated possible changes in BSR using both the data from females who were followed continuously for at least a year (Fertility data set; see Materials and Methods), and also all mothers for whom the sex of their offspring was known in the 28 years of the study (Biography data set). In the smaller data set, the BSR switched from female- to male-biased through time, decreasing from 67% to 34% females between 1987 and 2010 (*N* = 150 births to 37 mothers, β_1_ = 0.058, *P* = 0.032; Table 4, Figure 5). There was a non-zero estimate of the female-to-female variance in BSR, although this was not significant (σ^2^_m_ = 0.004, P = 0.065). If this non-significant term is removed from the analysis, the change in BSR through time is statistically stronger (β_1_ = 0.061, P = 0.017). A similar decrease was found in the larger Biography data set (*N* = 324 births to 94 mothers, β_1_ = 0.043, *P* = 0.036); although there was no effect of mother age, this analysis detected statistically significant variation in BSR among mothers (σ^2^_m_ = 0.039, P = 0.021; Table 4). Similar shifts to male-biased BSRs with increasing density have been observed in other primates [22], [23], although explanations of the mechanisms involved in such adjustments in BSR are still speculative [24].

**Table 4:** Effect of time on BSR for northern muriquis at the Reserva Particular do Patrimônio Natural Feliciano Miguel Abdala, Brazil from 1983 to 2010 (equation 4).

	Fertility data			Biography data		
Parameter	Value	Score^1^	P	Value	Score^1^	P
β_0_	−0.11			0.17		
β_1_ (time)	0.058	−2.15	0.032	0.043	2.10	0.036
σ^2^_m_ (mother-to-mother variation)	0.004	2.3	0.065	0.39	4.2	0.021

1 For fixed effects this is the z-score, and for random effects this is (χ^2^_0_+χ^2^_1_)/2. Statistical tests are only provided for those quantities of interest. For the Fertility data set, *N* = 150 observations from 37 mothers over 24 years in 4 age classes. For the Biography data set, *N* = 324 observations from 94 mothers over 28 years. For the Fertility data set, the effect of mother age class was not statistically significant (LLR χ^2^_3_ = 1.12, P>0.5).

We considered whether the combination of the increase in BSR (from 33% to 66% males) and the shorter average interbirth interval of males compared to females (1044 versus 1137 days, respectively; Table 2), might explain the decrease in interbirth intervals through time. If the decrease in interbirth intervals was caused solely by the change in BSR, the decrease in interbirth intervals would have been from 1106 days ( = 0.33*1044+0.67*1137) to 1076 days ( = 0.66*1044+0.34*1137), much smaller than the observed decrease from 1201 to 1015 days from 1987 to 2010. Because changes in the BSR had so little effect on the interbirth interval, BSR could not explain the increase in fertility exhibited in the population through time (Figure 1).

### Population Growth Rates

**Figure 6:**
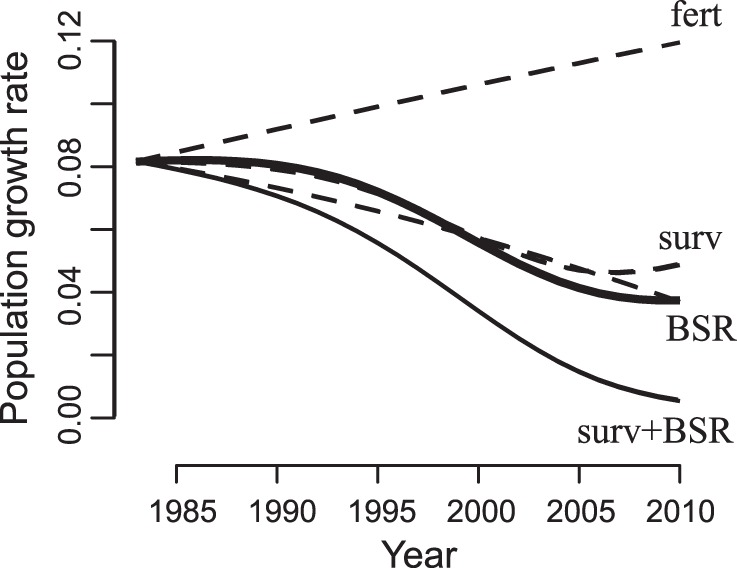
Per capita population growth rates computed from life tables separating effects of time-dependent demographic rates for northern muriquis at the Reserva Particular do Patrimônio Natural Feliciano Miguel Abdala, Brazil, 1983–2010 (See Materials and Methods). The thick solid line is the best prediction, using time-dependent fertility, survival, and BSR. The dashed line labeled “fert” is the case when only fertility changes; the dashed line labeled “surv” is the case when only survival changes; the dashed line labeled “BSR” is the case when only BSR changes; and the thin solid line labeled “surv+BSR” is the worst-case scenario when both survival and BSR change, but the fertility rate does not increase.

To reveal the relative impacts of the demographic changes on our study population, we computed the per capita population growth rate (PGR) from 1983 to 2010 while allowing or disallowing the observed changes in fertility, survival, and BSR (Figure 6). This was done by constructing a demographic projection matrix from the time-varying estimates of vital rates and calculating the asymptotic PGR for each year of the study (see Materials and Methods). For the 1983 demographic rates, the PGR is 0.082 yr^−1^, which corresponds to a population doubling time (PDT) of about 8 years. By 2010 the PGR had dropped to 0.037 yr^−1^ (PDT of 19 yr). Rather than steadily dropping, however, the rate of decrease of the PGR has begun to abate as the initial increase in young mortality has leveled off (Figure 3). If the survival rate and BSR had remained unchanged, the observed increase in fertility would have produced a PGR of 0.120 yr^−1^ in 2010 (PDT of 6 yr). The decreases in survival and BSR by themselves (i.e., keeping the two other demographic rates fixed) would have led to PGRs of 0.049 yr^−1^ and 0.037 yr^−1^ (PDTs of 14 and 19 yr), respectively. These findings show the effects of fertility, survival, and BSR on PGR were of similar magnitude. If fertility had remained fixed while both survival and BSR showed their observed declines, by 2010 the PGR would have been 0.005 yr^−1^ and the population would have nearly stabilized.

**Figure 7:**
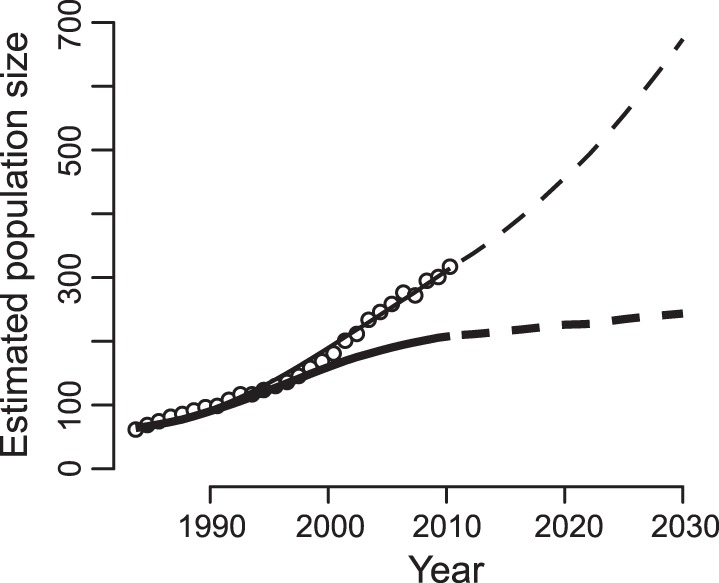
Estimated population size from 1983 to 2010 and projected population sizes to 2030 for northern muriquis at the Reserva Particular do Patrimônio Natural Feliciano Miguel Abdala, Brazil. The estimates of population size (dots) were obtained by total population counts after 2003. For the period before 2003, we estimated the population size using the estimated age of animals when they were first included in the population monitoring. For adults, only minimum and maximum estimates of age were used. In these cases, we assumed that their true age was uniformly distributed between the minimum and maximum age. Dots represent mean population estimates from 100 randomly constructed data sets. The upper line gives the estimated population size starting at the initial population estimate and projected through time using the life table constructed from time-dependent estimates of fertility, survival, and BSR. The correspondence between the dots and the line indicates that the demographic rates estimated from a subset of individuals in the population (line) correctly capture the population growth. After 2010 (dashed black line) the population was projected assuming that demographic rates were fixed at their 2010 values. The thicker, lower line is similar, yet assumes that there was no change in fertility from its 1983 estimated value.

To explore the consequences of changing demographic rates on the population within this forest fragment, we estimated population growth from 1983 to 2010 and then projected the population size to 2030 under two scenarios: (i) demographic rates changed as observed over the period 1983–2010 and then remained at their 2010 values, and (ii) fertility remained at its 1983 level, while survival and BSR followed their observed changes until 2010 and then remained at their 2010 values (Figure 7). If fertility had not increased from 1983 to 2010, the population would now be stabilizing at around 200 individuals, just two-thirds of its current size. In contrast, for vital rates fixed at their observed 2010 values, the population is predicted to more than double between 2010 and 2030. These results show that increasing fertility has allowed our study population to escape the upper density limit that would have been imposed by the observed changes in survival and BSR.

## Discussion

Our study population of northern muriquis has increased 5-fold since 1983 and now constitutes 1/3 of the total population of this endangered species. Despite its apparent recovery, demographic and behavioral anomalies in this population indicate stresses; density dependent mechanisms that regulate populations are not operating as expected [25], [26]. As the population continues to grow, density dependence will undoubtedly increase, but whether this will take the form of a decline in fertility or a further increase in mortality is not clear. Until these mechanisms are functioning effectively and understood, any management efforts to further increase the size of the population would be ill-advised.

The two unexpected demographic patterns that we observed – the increase in fertility and recent increase in mortality of prime-aged individuals – likely involved behavioral shifts of the muriquis. With increasing population size, muriquis increased the time spent on the ground, expanding their vertical niche [27]. This has permitted them to exploit fallen fruit and perhaps other previously under-utilized or ignored foods. We suspect that this is the main explanation for the improved nutritional status of females underlying the increase in fertility; although we cannot exclude the possibility that resources in the forest have changed over 28 years, observational evidence points to changes in the muriquis’ use of resources rather than changes in the resources themselves [28]. Indeed, recent floristic analyses predict recruitment of some muriqui food trees may begin to decline [29]; this would have the opposite effect on fertility from that observed. Increased use of ground habitats may also explain the recent increase in mortality among prime-aged males (Table 3, Figure 4) who, compared to females, spent more time on the ground [27] where they would be exposed to greater risks from terrestrial predators and pathogens [30]–[33].

The most direct management strategy to alleviate current population stresses is expanding the area of forest habitat to reduce overcrowding that is presumably forcing terrestriality. Recent sightings of five adolescent females that had dispersed into some of the small, unprotected forest fragments outside the boundaries of the Reserve (updated from [34]) are consistent with the high PGR, which is producing these potential colonists. Although it is possible that very low levels of female dispersal into these fragments may have occurred prior to the onset of population-wide monitoring in 2003, the current crowding at high population density in the Reserve is likely to increase its occurrence. Establishing connectivity between these fragments and incorporating them into the protection of the Reserve would simultaneously facilitate female dispersal and colonization success while also relaxing constraints on the current population. The hyper-inflated fertility rates that our analyses have revealed are unlikely to be sustainable over the long-term, and expanding the Reserve’s area will provide both a pressure valve for demographic stress and a geographically expanded population that will likely be more resilient against unforeseen threats.

A broad conclusion about conservation of endangered species can be drawn from these findings: detailed information about demographic rates is essential for evaluating population health. While detailed demographic studies are typically performed for population viability analyses of species approaching extinction, recovering species with increasing population sizes do not typically generate the same degree of attention [7]. Important exceptions have demonstrated the critical role of intensive conservation efforts in local population recoveries [10], the value of distinguishing short-term versus long-term population responses in management decisions [35], and the risks of relying exclusively on indicators of density dependence in population management decisions [36]. Indeed, analyses of refuge design and management of recovering populations too often concentrate on determining the “carrying capacity” of reserves, which at least for our study population would have been predicted incorrectly due to the unexpected increase in fertility. Thus, analyses of demographic rates that identify the stresses on recovering populations not only add to the growing literature on the effects of plasticity in behavioral and life history traits on population demography [6], [37], [38], but are essential for the design of informed management tactics to ensure long-term population persistence.

## Materials and Methods

### Ethics Statement

This research involved non-invasive work with wild non-human primates. All work was done in accordance with guidelines of the national authorities where the work occurred.

### Data Sets

Two related data sets were used for the analyses. The “Biography” data set consisted of 463 records representing each individual animal included in the study as of December 2010 and was used to calculate mortality rates. The “Fertility” data set was a subset of the Biography data set that includes only females for which continuous observations were made for at least a year. All variables are described in Strier and colleagues [15]; details relevant to the present analyses are summarized here:

- Unique animal identification (ID) – There is 1 known female and up to 12 other females in the population who could potentially have 2 discontinuous IDs. These females dispersed or disappeared from the main study group, Matão, prior to 2003, and because during this time no censuses were taken on the other 3 groups, the females originally from the Matão group could have joined one of the other groups and then entered the data set with a new ID in 2003. This will not affect our estimates of demographic rates, although it may affect estimates of total population size (see “Projections of population size”, below).
- Sex if known - The sex was known for all but 3 infants.
- Observed first entry date into the data set.
- Type of entry – Entry types distinguished births from first observations of non-infants.
- Estimated birth date (BD), along with a minimum and maximum BD estimate – Because muriquis are long-lived, the BD of individuals is uncertain if they were older than infants when they first entered the data set. For these individuals, age was estimated at first observation and the BD thereby estimated. Due to the uncertainty in determining age, the minimum and maximum BD were assigned assuming that adults could be between 10 and 30 years old.
- ID of the mother if known – The mother ID was known for 337 individuals.
- Population group of birth if known – The total population contains 4 groups, with observations conducted on only the main group, Matão, starting in 1983. The study was expanded to all groups by 2003.
- Departure date from the data set – Individuals who were still in the population at the end of the data set were recorded as such.
- Type of departure – Departure type included confirmed death (129 individuals), loss from observations (16 individuals), dispersal from the study population (13 females, including 10 that emigrated from their natal Matão group prior to 2003, when it was the only group in the study), and individuals remaining at the end of the data set.

The “Fertility” data set used in our analyses consists of information from 43 females in the Matão study group and covers the period 1987–2010; earlier years did not have sufficiently intensive sampling for inclusion in the data set. About a third of these females were re-sighted at approximately 1–3 day intervals (depending on their ranging patterns), and the others were re-sighted during monthly sampling. We used this high-quality data set for estimating fertility rates to err on the side of caution; the continuous observations eliminate potential missed births resulting from observation gaps of a few weeks, when a birth/death could have been missed.

### Fertility

Changes in fertility through time were analyzed using the Fertility data set. One set of twins occurred in the data set, and this was treated as a single birth event. Data were fit to a Generalized Linear Mixed Model (GLMM) [19], [39] assuming that whether or not a female gave birth in a calendar year, *B_i_*, was the outcome of a Bernoulli process:
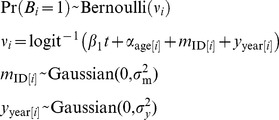
(1)


The coefficient β_1_ gives changes in the probability of giving birth through time (year *t*). We investigated dividing the females into 3, 4 or 5 age classes, and for each number of age classes, we investigated different partitions (under the restriction that each age class contains at least one female in each year). The age classification that gave the lowest value of Akaike’s Information Criterion (AIC) had age classes 7–11, 12–19, 20–29, and 30+ corresponding to young adult (i.e., based on youngest [8] and mean known age of first reproduction [20]), prime-aged, middle-aged, and old females. The model incorporates the possibility of variation among females in fertility by assuming female-specific fertility rates of *m*_ID[*i*]_ where the function ID[*i*] maps the datum *i* onto the identity of the mother; this accounts for repeated measures on the same individuals. Possible variation in the number of births per year affecting all females was incorporated into the random effect *y*_year[*i*]_ where year[*i*] maps the datum *i* onto the year of observation; this accounts for possible lack of independence among births caused by environmental variation.

This model was fit to data using the function lmer [40] in the R statistical programming language [41]. Statistical significance is provided for the fixed effects α*_j_* by z-scores and for random effects σ^2^ by likelihood ratio tests. Because the values of σ^2^ are constrained to be greater than zero, the significance levels are given by a 50∶50 mixture of χ^2^_0_ and χ^2^_1_ distributions, (χ^2^_0_+χ^2^_1_)/2 [42], [43]; this means that the P values calculated from an ordinary likelihood ratio test must be divided by 2.

### Interbirth Interval

In addition to estimating fertility rates, we investigated the interbirth interval as a possible finer measurement of fertility. If interbirth intervals decreased through time, this would imply an increase in fertility.

In the Fertility data set, there were records of 87 interbirth intervals for 28 females; this excludes interbirth intervals in which the first offspring died. Scoring the interbirth interval *I_i_* as the number of days between births, we fit the model
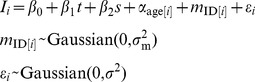
(2)where *t* is the date of the first birth, *s* is the sex of the first offspring, α_age[*i*]_ is the age of the mother using the same age classes as in the analysis of fertility, *m*_ID[*i*]_ accounts for variation among mothers in their interbirth intervals, and ε*_i_* gives residual variation. We also performed a similar analysis including interbirth intervals in which the first infant died within the calendar year of its birth, treating the death of this infant as a categorical variable in the model given by equation 2. Finally, we used the same approach to investigate the age at first reproduction (AFR) of females, including in the model the date of birth of both the mother and the offspring, as well as the sex of the offspring.

### Mortality

Mortality was calculated using the Biography data set based on the number of individuals with observed deaths on a yearly (calendar) basis. Thus, the analysis is based on annual data in contrast to mortality analyses based on the age-at-death of individuals. We took this approach because it gives estimates that can be used directly in life tables and because many individuals in the population were alive at the end of the study period. Furthermore, this approach allowed us to incorporate non-independence among individuals caused by year-to-year fluctuations in mortality that affected all age classes [20]. Our analyses use only recorded deaths (129 individuals) rather than also including those recorded as permanently missing (16 individuals); including those permanently missing does not change any of the results.

Data were fit assuming that the number of individuals in each age class dying in each year *t*, *Y_i_*, is given by a binomial distribution with *N* total individuals and probability of mortality *p_i_*:
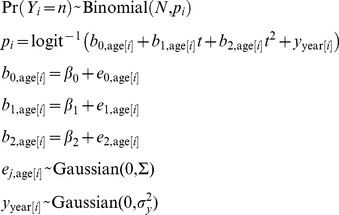
(3)


Here, the mortality probability *p_i_* is a quadratic function of time *t* to allow possible nonlinear changes in mortality over the course of the study period. The coefficients *b_j_*_,age[i]_ are assumed to be Gaussian random variables with mean β*_j_* that differ among age classes; the function age[*i*] maps the identity of data record *i* onto the individual’s age class at time *t*. The variances and covariances among the values of *b_j_*_,age[*i*]_ are given by the covariance matrix Σ. In addition to the quadratic effect of time on mortality, we included random variability *y*_year[i]_ that incorporates variation in mortality among years which affects all age classes in the same way; including the term *y*_year[*i*]_ accounts for the lack of independence among mortality events within the same year that could be caused by, for example, extreme weather events. Diagnostic plots of the model residuals validated the adequacy of the quadratic model to capture changes in mortality through time (e.g., to exclude the possibility of threshold changes in mortality through time).

Because age was treated as a random effect, there is no loss of “degrees of freedom” caused by partitioning individuals into finer age classes. Therefore, we selected the finest partition that guaranteed at least one individual in each age class in each year based upon their estimated BD; the 15 age classes were 0, 1, 2, 3, 4, 5, 6, 7, 8–9, 10–14, 15–19, 20–24, 25–29, 30–34, and 35+ where, for example, individuals in age class 0 were born in that year, individuals in age class 1 were born in the preceding year, etc. When age classes occur in the terms *b*_1,age[*i*]_ *t* and *b*_2,age[*i*]_ *t* ^2^, they act in the same way as interaction effects between *t* (or *t* ^2^) and age. For example, a significant value of the variance of *e*_2,age[*i*]_ means that *b*_2,age[*i*]_ varies significantly among age classes; therefore, age classes differ the quadratic effect of time on mortality. To test for the statistical significance of *b*_2,age[*i*]_, the likelihood ratio test involves removing two terms from Σ, the variance of *e*_2,age[*i*]_ and its correlation with *e*_1,age[*i*]_, so the resulting test statistic is (χ^2^_0_+2χ^2^_1_+χ^2^_2_)/4 [42], [43].

We treated age class as a random effect in the test for whether mortality changes through time, because this avoids possibly overparameterizing the model if age class were treated as a fixed effect. If treated as a fixed effect, age classes will lead to 45 separate coefficients, in contrast to 9 parameters when treating age as a random effect (coefficients β_0_, β_1_, β_2_, and the elements of Σ). Nonetheless, for estimating mortality for the demographic projection matrix, we treated age class as a fixed effect, because this does not restrict the estimates of the coefficients to follow a Gaussian distribution. Our concern about overparameterization occurs because when treating age class as a fixed effect, the quadratic term *b*_2,age[i]_ *t* ^2^ in equation 3 is more highly statistically significant (LLR, χ^2^_14_ = 37.2, P = 0.0007) than when treating age classes as a random effect (LLR, χ^2^_0_+χ^2^_1_ = 8.2, P = 0.006), even though there is a greater loss of degrees of freedom when treating age classes as fixed effects. We report the statistical tests when treating age classes as a random effect because they are more conservative (have higher P values) and note that the results from the demographic projection matrix change little between treating age classes as fixed vs. random effects.

### Birth Sex Ratio

Changes through time in BSR were estimated using both the Fertility and Biography data sets. Sex was scored as female = 0 and male = 1, and the sex of offspring *i*, *S_i_*, was modeled as the outcome of a Bernoulli process:
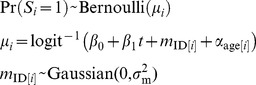
(4)


The coefficient β_1_ gives changes in the BSR through time (year *t*). The model incorporates the possibility of variation among females in BSR by assuming that the sex of offspring is given by *m*_ID[*i*]_ where ID[*i*] maps the datum *i* onto the identity of the mother. In the Fertility data set we also investigated possible variation in BSR according to mother age, incorporating the fixed effect α_age[*i*]_ where age[*i*] maps the datum *i* onto the age of the mother. For this analysis, mother age classes of 7–11, 12–19, 20–29, and 30+ were used to match the age classes in the fertility analyses.

### Per Capita Population Growth Rates

We constructed a time-dependent demographic projection matrix model based on life-table data available at the individual level in the studied population for mortality, BSR, and fertility. This matrix is equivalent to that of Morris and colleagues [20] with the exception of our inclusion of time-dependent changes in vital rates and our partitioning of individuals into finer age classes than were allowed by the comparative data sets analyzed there. From the demographic projection matrix we then computed the asymptotic per capita population growth rate (PGR).

The demographic projection matrix *L*(*t*) corresponds to a pre-breeding census model with full age dependence:
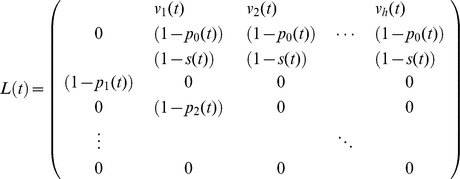
where *p_a_*(*t*) is the mortality of females of age *a* in year *t*, *v_a_*(*t*) is the fertility of females of age *a* in year *t*, and *s*(*t*) is the BSR; because females are never mate limited, *L*(*t*) gives the population growth in terms of the female population. Values of *p_a_*(*t*), *v_a_*(*t*), and *s*(*t*) were those estimated from the data as described above.

The PGR is given by log(eig *L*(*t*)), where eig *L*(*t*) is the dominant eigenvalue of *L*(*t*) [18]. To assess the importance of changes in the different demographic rates, we also computed the PGR while assuming that mortality, BSR, and/or fertility did not change through time but were instead fixed at their estimated 1983 values (Figure 6). Note that log(eig *L*(*t*)) is the per capita population growth rate that would be achieved by a population at the stable age distribution. Because the demographic rates are changing through time, the population will not be at the stable age distribution. We also calculated population growth rates for the time-varying *L*(*t*) directly by simulating population growth to account for transients away from the stable age distribution; these differed quantitatively only slightly from the rates calculated from log(eig *L*(*t*)). We chose to use log(eig *L*(*t*)) to depict the PGR because this provides an unambiguous comparison among the scenarios in which mortality, BSR, and/or fertility are set at their 1983 estimates. Specifically, in each of the different scenarios, the departure of the initial observed age distribution from the stable age distribution will be different. Therefore, the effects of transient dynamics will differ among scenarios. In contrast, the PGR based on log(eig *L*(*t*)) can unambiguously be interpreted as the asymptotic population growth rate that would be achieved if the demographic rates were fixed at *p_a_*(*t*), *v_a_*(*t*), and *s*(*t*) at time *t.*

### Projections of the Population Size

A challenge in estimating population size is that only one group within the population was initially observed and censused; the entire population was only censused starting in 2003. The population size can be backcast from 2003 using the age of the animals, but ages of adults when they were first observed and entered into the data set were only estimated approximately. To overcome this, we used the following procedure. For each year starting in 1983, we determined the size of the population as the number of individuals in the Biography data set who had birth dates on or preceding that year and death dates after that year. For individuals for which the minimum and maximum estimates of birth year were not the same, we assumed that the true birth year was randomly distributed uniformly between these estimates. We then computed the population size. We repeated this procedure 100 times and took the average of the 100 estimates to give the population trajectory (circles in Figure 7).

Our estimate of 60 individuals for the initial population size in 1983 was higher than the 40–45 individuals estimated from 1982 census data [44]. This discrepancy might in part be explained by multiple IDs being assigned to the same females in the data set. Ten females who were last observed traveling with a non-natal group prior to the onset of the Reserve-wide monitoring in 2003 were scored as having dispersed; two other females old enough to disperse disappeared during this period and were scored as permanent disappearances. These females could not be re-identified and may have entered the data set under new IDs, with estimated BDs and unknown histories, when monitoring of the other groups was initiated. Assuming all of these females survived to be resurveyed with new IDs, our estimate of the 1983 population could be as low as 48. This, however, will not affect our overall conclusions and the rate of population growth.

To compare with the population projections from the life table *L*(*t*), for each of the 100 simulations we started the population at its estimated size in 1983 and then projected the population forward using *L*(*t*) to describe female numbers; males were included using the estimates of BSR (Table 4) and male mortality (equation 1, but treating coefficients for different ages as fixed effects). The 100 projections were then averaged to give a projection (line in Figure 7) to compare to the average estimates of population size.

Although the same data were used for both the estimates of population size and population projections using the life tables, their close match supports the statistical analyses of the changing demographic rates. The analyses of the Biography data set, and the Fertility subset, to give demographic rates involve numerous assumptions about the structure of the data (e.g., whether demographic rates depend on age) and the functions (e.g., whether mortality is a quadratic function of time). The fit between the resulting population projections and the estimates of population size shows that the processes captured by the demographic analyses are sufficient to explain the changes in population size.
